# Microcephalic Osteodysplastic Primordial Dwarfism Type II With Associated Glucose-6-Phosphate Dehydrogenase Deficiency in a Saudi Girl

**DOI:** 10.7759/cureus.19829

**Published:** 2021-11-23

**Authors:** Fadi Busaleh, Haider Alnofaily, Hussain A Al Ghadeer, Fatimah A Albahrani, Hibah A Alatiyyah, Salwa B Alshaikh, Ahmed M Alhamrani, Walaa Hassan, Jumanah Alatiya, Jawad Alnaqaa

**Affiliations:** 1 Pediatrics, Maternity and Children Hospital, Al-Ahsa, SAU; 2 College of Medicine, King Faisal University, Al-Ahsa, SAU; 3 Family Medicine, King Faisal University, Al-Ahsa, SAU; 4 Pediatrics Department, Saudi Ministry of Health, Al-Ahsa, SAU; 5 Surgery, Al Kharj Military Industries Corporation Hospital, Al Kharj, SAU

**Keywords:** microcephalic osteodysplastic primordial dwarfism type 2, glucose-6-phosphate dehydrogenase deficiency, dwarfism, intrauterine growth restriction, microcephaly

## Abstract

Microcephalic primordial dwarfism is a group of disorders that result in growth restriction and multiple morbidities. The condition is subdivided into three categories, with microcephalic osteodysplastic primordial dwarfism type II (MOPDII) being the most prevalent. Globally, only a few cases have been reported, with only available information about these disorders described in the literature. In this case report, we present the clinical findings seen in an infant with MOPDII in Saudi Arabia with associated glucose-6-phosphate dehydrogenase deficiency hemolytic anemia.

## Introduction

Microcephalic osteodysplastic primordial dwarfism type II (MOPDII) is an autosomal recessive multisystemic disorder caused by mutations in the *pericentrin* gene (*PCNT* variant c.6366_6367del). The disorder presents with several features, including microcephaly (a small-sized head), short stature with stunted growth, skeletal dysplasia, tooth abnormalities, distinctive facial features, and neurovascular angiopathy [1]. Morbidities associated with MOPDII are variable. Hall et al. reported several cases of this condition complicated by cerebrovascular diseases such as stroke or moyamoya disease, cognitive decline, chronic kidney disease, hypertension, growth retardation, or diabetes mellitus [1]. Although MOPDII is very rare with an unknown prevalence, it is considered to be one of the most common forms of microcephalic primordial dwarfism (MPD). To date, globally, more than 150 cases have been reported [2]. As such, because the literature available regarding this disorder is limited, it is yet to be fully elucidated. In Saudi Arabia, in particular, there is a scarcity of published literature on this condition, with only three cases of MOPDII reported to date [3,4]. In this case report, we highlight the prospects of MOPDII in light of previously documented literature and its association with glucose-6-phosphate dehydrogenase (G6PD) deficiency hemolytic anemia.

## Case presentation

A 12-month-old girl was diagnosed with MOPDII through a genetic study that showed homozygous pathogenic variants in the *PCNT* gene associated with homozygous pathogenic variants in the *G6PD* gene, resulting in G6PD deficiency hemolytic anemia.

**Figure 1:**
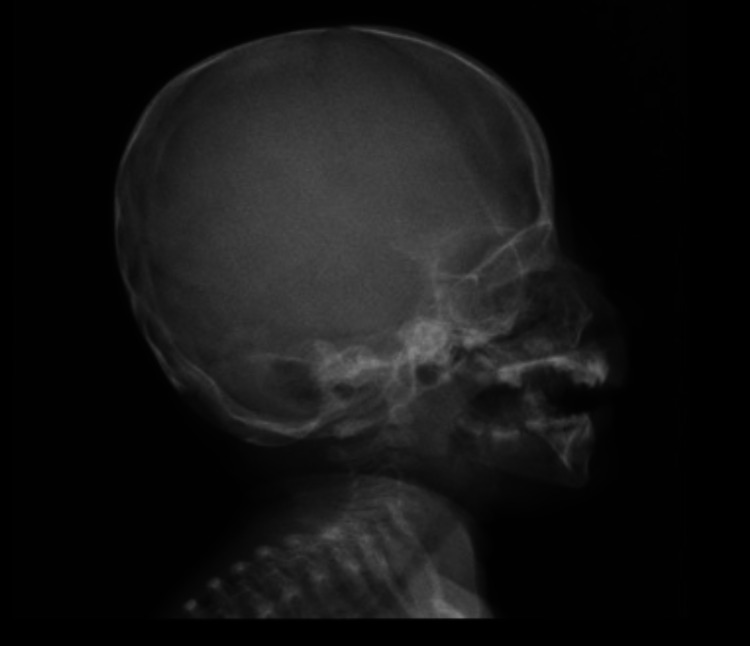
X-ray of the head: lateral view showing normal skull shape with no deformity; however, it is smaller in size for her age.

**Figure 2:**
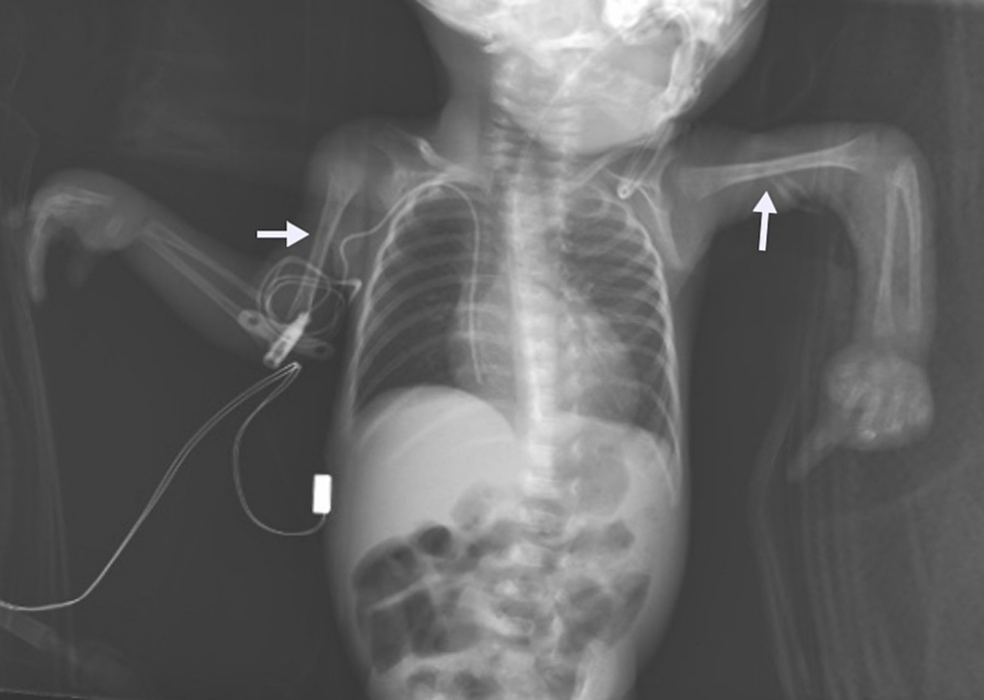
X-ray of the upper body (part of the skeletal survey): anteroposterior view showing shortness of the middle portion of upper limps (mesomelia) (white arrows).

**Figure 3:**
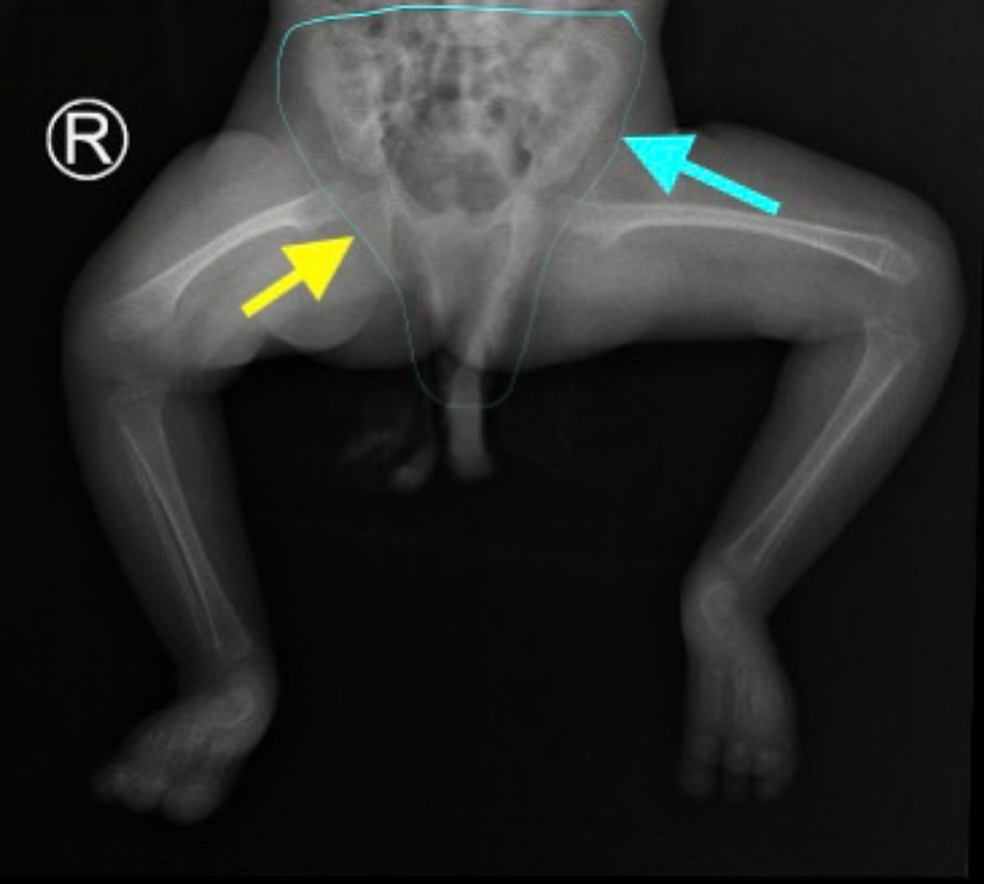
X-ray of the lower limbs (part of the skeletal survey): anteroposterior view showing a high and narrow hip (blue arrow and line) and a flat acetabulum (yellow arrow).

The patient was the second child of a second-degree consanguineous marriage of two healthy parents with no similar condition in their family. The pregnancy was normal till 20 weeks of gestation when regression of fetal growth was noted on sonography. The mother was screened for possible risk factors such as preeclampsia, TORCH infections, and placental disorders, which were negative. Due to antenatal growth restriction, she was delivered prematurely at 34 weeks with severe intrauterine growth restriction (IUGR). At birth, her growth parameters were below the third centile for her age (she weighed 620 g, had a head circumference of 23 cm, and was 30 cm long). She was nursed in the neonatal intensive care unit (NICU) for 126 days for weight gain, treatment of gram-negative sepsis, and control of idiopathic hypertension, which resolved upon discharge. However, her dysphagia persisted after discharge from the NICU. Hence, she was maintained on nasogastric feeding tubes. Because of feeding issues, she developed aspiration pneumonia twice during swallowing training, which led to hospitalization during both episodes. A videofluoroscopic swallowing study was performed which was normal with no evidence of regurgitation or incoordination of food bolus (Video 1). Currently, the patient is off nasogastric feeding tubes as she is successfully tolerating oral feeds. She has characteristic facial features that were subtle during infancy but became clear over time, including a peaked nose with a broad nasal bridge, a small jaw, and full cheeks; however, her ears appear to be normal in size and shape with normal orientation. According to her latest growth assessment, she gained weight and grew, but her values have remained below the third centile for her age (weight of 2.5 kg, head circumference of 34 cm, and length of 49 cm). A skeletal survey was performed which showed shortness of the middle portion of her long bones (mesomelia) with a narrow hip, and her skull was smaller in size for her age (Figures 1-3). Echocardiography demonstrated a small restrictive ventricular septal defect and patent ductus arteriosus, which closed at the age of six months during her cardiology follow-up, with no need for any medications. The patient’s development is normal for her age, as she has begun to crawl, sit without support, and say “baba.” Finally, as she does not have any active issues currently, she is on regular follow-up with general pediatric and endocrinology for assessment of growth, orthopedics for follow-up of skeletal dysplasia, and pediatric neurology for follow-up of cognition and any anomalies.

**Video 1:** Videofluoroscopic swallowing study showing normal coordination and formation of bolus with no regurgitation and normal propagation of barium to the esophagus without any signs of anatomical obstruction.

## Discussion

Fetal growth restriction is a term used when a fetus fails to achieve the expected intrauterine growth, which is defined as a birth weight below the 10th centile, with severe restriction referring to birth weight below the third centile [5]. Although restriction of fetal growth can be due to numerous factors, it is mainly related to three basic defects, namely, the genetic background of the fetus, maternal illness, or placental disorders [6]. MPD is one of the rarely encountered genetic causes of fetal growth restriction. It can result in a spectrum of complications that begin early in intrauterine life and continue throughout life. MPD is subdivided into three groups: I, II, and III. Type I and III are variants of a known disorder characterized by cephalo-skeletal dysplasia, as described by Drs. Linder and Tybi. While the second type of MPD, MOPD II, is characterized by global growth restriction and microcephaly [7].

MOPDII is characterized by different patterns of growth restriction with multisystemic involvement [1]. Growth restriction is the cardinal feature that begins antenatally as the fetus shows decompensation of growth early in the first and second trimesters which can be complicated by prematurity [1]. This results in a neonate born prematurely with IUGR and its complications. Examples of such complications include neonatal sepsis, metabolic and electrolyte disturbance, prolonged NICU admission, and idiopathic hypertension, among others [1,8,9]. This is consistent with our patient who required NICU admission and developed multiple complications due to prematurity and IUGR.

Skeletal dysplasia and bone formation anomalies are the secondary complications of MOPDII. The disorder can affect any bony part of the body, ranging from head to toe. Microcephaly is the first skeletal anomaly, as the syndrome name is derived from it, followed by shortening and thinning of long bones, which lead to mesomelia, mainly in the upper limbs, along with widening of the metaphysis. In addition, hip anomalies are observed, including narrow and high pelvis at birth, which can progress to coxa vara. Other anomalies are also seen such as lateral dislocation of knees, scoliosis, and delayed ossification of bones [10]. Furthermore, teeth, particularly secondary teeth, can be impacted and become smaller and more dysplastic, resulting in enamel hypoplasia, with teeth loss occurring naturally by the third decade [1]. As our patient is still in infancy, she suffers from microcephaly, mesomelia with a small hip, and stunted growth. Her teeth are normal currently as they are primary teeth. Future appointments with a pediatric orthopedic, pediatric endocrinologist, and dentist are needed as other dysplastic features are likely to become more visible as she grows.

Neurovascular injuries are the most common cause of morbidity and mortality in MOPDII, with an estimated lifetime percentage reaching up to 50%. The main angiopathic phenomena include aneurysms and moyamoya disease (collateral vessels developed in response to obstructed large arteries in the brain). Therefore, screening for these angiopathies is recommended. Some recommend starting in the first year of life and continuing yearly, while others recommend starting after the second year of life and following up every other year. The gold standard screening tests include magnetic resonance imaging of the brain with magnetic resonance angiography (MRA) of the great arteries in the brain, including the circulus arteriosus cerebri (circle of Willis), major cerebral, and cervical arteries. Neurological manifestations are variable, ranging from a decline in cognitive function to a full motor or sensory neurological deficit. Each angiopathic phenomenon has its unique management option. In the case of moyamoya disease, revascularization and restoration of normal blood flow are the treatment goal, while removal of the distended part is the treatment goal for aneurysms [11,12]. Our patient is currently 12 months old and is scheduled for neurological follow-up with MRA.

Although MOPDII patients have normal intellectual development compared to their peers, sometimes there is a decrease in intellectual development secondary to brain insult from neurovascular events [12]. This emphasizes the importance of regular neuroimaging screening.

Prolonged dysphagia can be noted in MOPDII patients and leads to a prolonged assessment of tube feeding techniques. In our patient, as the videofluoroscopic swallow study was normal, there was no evidence of functional, anatomical, or neurological causes of feeding difficulties [1]. Hence, practical feeding technique issues are the most likely cause of delayed oral feeding tolerance.

Cardiovascular complications such as septal or valvular defects can be seen after birth or later during adolescence as cardiovascular vessel obstructive disease [1,11]. This is consistent with our case as the patient had a ventricular septal defect with patent ductus arteriosus but did not require any interventions as they were restrictive and are expected to close spontaneously, according to the cardiologist.

Further, insulin resistance can develop in MOPDII, resulting in type II diabetes mellitus. It usually develops by the middle of a patient’s second decade of life [2], mandating regular follow-up with an endocrinologist.

Renal abnormalities and essential hypertension are seen in MOPDII, although with a lower percentage than other complications. In addition, some dermatological manifestations are also noted such as hypo- or hyper-pigmented lesions, for example, *cafe-au-lait* spots. Finally, the hematological system is the last to be involved, resulting in asymptomatic thrombocytopenia, anemia, or leukocytosis [1].

Clinical features and radiographic changes are used for diagnosis, which is confirmed by a homozygous genetic mutation in the *PCNT* gene located on the long arm of chromosome number 22. It is inherited in an autosomal recessive pattern, so both parents carry the defective gene [1]. This is consistent with our patient as she had a deletion mutation in the *PCNT* gene (variant c.6366_6367del), along with characteristic clinical features and initial radiographic findings of MOPDII. Moreover, she had a mutation on the X chromosome (*G6PD* variant c.653C>T) that resulted in a subtype of hemolytic anemia (G6PD deficiency), which added to the anemia caused by MOPDII. To our knowledge, this is the first case of MOPDII with concurrent G6PD deficiency.

G6PD deficiency hemolytic anemia by itself is a benign condition that can be prevented by avoiding oxidative stress by ingestion of fava beans or sulpha drugs. Our patient needs to be on a diet that avoids oxidative stressors [13]. Furthermore, MOPDII patients are at risk of iron deficiency anemia, with low hemoglobin levels. Hence, any further drop in hemoglobin, especially with hemolytic crises caused by G6PD deficiency hemolytic anemia, can cause significant hemodynamic instability.

## Conclusions

Management of patients with MOPDII dwarfism requires a multidisciplinary team. Like any other disease, it is associated with several complications, but some complications need more attention than others. Neurovascular anomalies are the most significant complications which can be fatal if left unnoticed as hemorrhagic or ischemic strokes can occur. In addition, skeletal dysplasia is a significant but not a deforming complication, except when associated with scoliosis. A management plan including regular growth assessment with neuroimaging is crucial for early detection of complications and early interventions. Finally, concurrent G6PD deficiency places patients at higher risk of oxidative stress and worse anemia; hence, patients need to follow a special diet with instructions given to the family about oxidative stress and how to avoid it.
